# A Novel Classification of Lung Cancer into Molecular Subtypes

**DOI:** 10.1371/journal.pone.0031906

**Published:** 2012-02-21

**Authors:** Lisandra West, Smruti J. Vidwans, Nicholas P. Campbell, Jeff Shrager, George R. Simon, Raphael Bueno, Phillip A. Dennis, Gregory A. Otterson, Ravi Salgia

**Affiliations:** 1 CollabRx Inc., Palo Alto, California, United States of America; 2 Department of Medicine, Section of Hematology/Oncology, The University of Chicago, Chicago, Illinois, United States of America; 3 Symbolic Systems Program (Consulting), Stanford University, Stanford, California, United States of America; 4 Department of Medicine, Section of Hematology/Oncology, Medical University of South Carolina, Charleston, South Carolina, United States of America; 5 Division of Thoracic Surgery, Brigham and Women's Hospital, Boston, Massachusetts, United States of America; 6 National Cancer Institute, Bethesda, Maryland, United States of America; 7 Ohio State University Comprehensive Cancer Center, Columbus, Ohio, United States of America; University of Nebraska Medical Center, United States of America

## Abstract

The remarkably heterogeneous nature of lung cancer has become more apparent over the last decade. In general, advanced lung cancer is an aggressive malignancy with a poor prognosis. The discovery of multiple molecular mechanisms underlying the development, progression, and prognosis of lung cancer, however, has created new opportunities for targeted therapy and improved outcome. In this paper, we define “molecular subtypes” of lung cancer based on specific actionable genetic aberrations. Each subtype is associated with molecular tests that define the subtype and drugs that may potentially treat it. We hope this paper will be a useful guide to clinicians and researchers alike by assisting in therapy decision making and acting as a platform for further study. In this new era of cancer treatment, the ‘one-size-fits-all’ paradigm is being forcibly pushed aside—allowing for more effective, personalized oncologic care to emerge.

## Introduction

Lung cancer kills more patients than any other malignancy in the world [1]. Early-stage disease can be treated with curative intent although the risk for relapse is notoriously high. Unfortunately, the majority of lung cancer patients present at an advanced stage. Despite an initial response to treatment, most of these late stage patients will eventually progress on standard therapy and die from their disease [2]. Despite the complex nature of lung cancer biology, its molecular underpinnings are becoming increasingly clear [3]. The discovery of a number of these molecular alterations underlying lung cancer has led to uniquely targeted therapies with specific inhibitor drugs such as erlotinib and gefitinib for mutations in the epidermal growth factor receptor (EGFR) [4], [5] or crizotinib for the gene translocation resulting in the EML4-ALK oncogene [6].

We have previously developed a formal process for classifying a cancer - melanoma - into molecular subtypes [7]. Molecular subtypes are defined as those tumors containing the same set of molecular (primarily genetic) defect(s) and their associated pathways. The division of a cancer into subtypes is purposeful in that each subtype has proposed treatment guidelines that include specific assays, targeted therapies, and clinical trials. This process produces a formal ‘molecular disease model’ that can be used by clinicians to guide treatment decisions, and refined by researchers based on clinical outcomes and laboratory findings.

**Figure 1:**
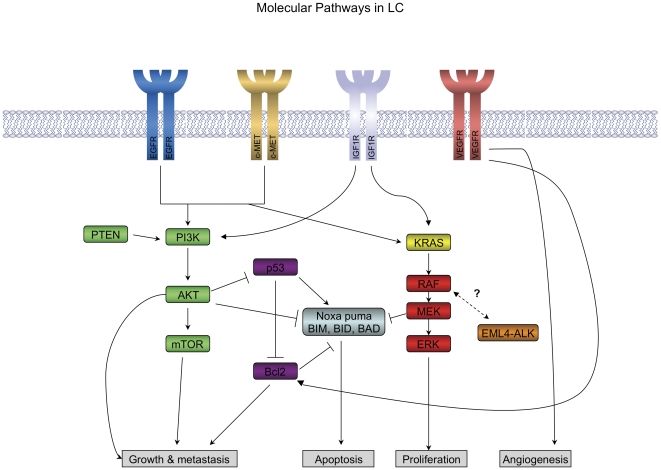
A major signaling pathway implicated in lung cancer is the EGFR pathway which signals to both the AKT/PI3K pathway (green) and the MAPK pathway (red) which regulate cell growth, proliferation and cell death. There is significant cross-talk between these pathways and their downstream effectors, which we have classified into 6 pathways for simplicity to account for differences in treatment modalities. The additional 4 pathways are: EGFR (blue), KRAS (yellow), EML4-ALK (orange), and P53/BCL (purple). It is thought that the RAS/RAF/MEK/MAPK pathway may be constitutively activated by the EML4-ALK fusion oncogene [100]. The complex relationship between this pathway and EML4-ALK is indicated with a dashed line.

In light of the growing insight into the molecular mechanisms underlying lung cancer with the development of sophisticated molecular diagnostics and targeted therapies, we now extend the molecular subtyping approach to lung cancer. Similar to the previously described melanoma molecular disease model, the lung cancer molecular disease model consists of a set of actionable molecular subtypes and proposed practice guidelines for treating each subtype. In contrast to the melanoma model, there is a larger molecular heterogeneity that exists within lung cancer (see Figure 1). Therapies (approved or experimental) that should be considered and those that are contraindicated are discussed. A subtype is deemed actionable if there is both an approved assay to determine whether a given tumor fits that classification and at least one FDA-approved or experimental targeted therapy with potential efficacy for that subtype. An example would be lung tumors containing the EGFR exon 19 mutation for which commercial assays and targeted agents are currently available. The latest version of this model can be found online here: <http://bigmac.collabrx.com/lc_edit/index.php/A_Lung_Cancer_Molecular_Disease_Model>.

The online version contains additional in-depth information about relevant genes, genetic tests, pathways, drugs, targets, and clinical trials, all hyperlinked and organized in a Wikipedia-like format. Given the evolving state of knowledge, we anticipate this baseline model will need to be revised routinely with new clinical and scientific findings. Existing types are likely to be split into new subtypes corresponding to responders and non-responders, and new types are likely to be added to accommodate previously unseen tumor groups. Over time, this model will be defined with greater and greater specificity and linked to increasingly efficacious therapies.

## Results

Tables 1 and 2 summarize the subtypes of lung cancer, roughly in order of importance of the associated oncogene/tumor suppressor, prevalence and potential for therapeutic intervention. The oncogenes that define the subtypes in Table 1 are currently high ‘strength of evidence’ (SOE) and capable of serving as the dominant oncogene and putative point of intervention for therapy, whereas the oncogenes and tumor suppressor genes that define subtypes in Table 2 are medium or low SOE and “on the horizon” for lung cancer treatment strategies. Figure 1 shows the major signaling pathways implicated in lung cancer. It is our hope that the present model serves to focus translational research on issues that may directly impact patient care, and that the resulting activity will lead to updates in the model.

**Table 1:** Principle lung cancer molecular subtypes.

Sub-type	Description	Pathway	Potentially relevant therapies	Relevant histological subtypes	Strength of evidence for clinical use*
1.1	EGFR sensitizing mutations	EGFR	TKIs & chemotherapy	Adenocarcinoma	High
1.2	EGFR resistance mutations including T790M	EGFR	Dual EGFR/HER2 TKI, c-MET inhibitors +/− 1^st^ or 2^nd^ generation EGFR TKIs, Hsp90 inhibitors, dual MET/VEGFR2 inhibitors, Chk1 inhibitors	Adenocarcinoma	High
1.3	VeriStrat proteomic signature	EGFR	TKIs & bevacizumab	Adenocarcinoma	High
2.1	K-ras mutations	K-ras	Dual MAPK & AKT/PI3K inhibitors, Hsp90 inhibitors	Adenocarcinoma	High
3.1	EML4-ALK	EML4-ALK	ALK inhibitors, Hsp90 inhibitors	Adenocarcinoma	High

**Table 2:** Secondary lung cancer molecular subtypes.

Sub-type	Description	Pathway	Potentially relevant therapies	Relevant histological subtypes	Strength of evidence for clinical use*
4.1	c-MET overexpression	c-MET	c-MET inhibitors, Dual Met/VEGFR2 inhibitors, ALK/MET inhibitors, c-MET monoclonal antibodies	Adenocarcinoma, small cell carcinoma, squamous	Medium
4.2	c-MET mutations	c-MET	c-MET inhibitors, dual Met/VEGFR2 inhibitors, ALK/MET inhibitors, c-MET monoclonal antibodies	Adenocarcinoma, squamous, large cell, small cell carcinoma	Low
5.1	PI3KCA amplification, mutations	AKT/PI3K	PI3K, AKT, mTOR inhibitors	Adenocarcinoma	Low
5.2	PTEN deletions/methylation	AKT/PI3K	PI3K, AKT, mTOR inhibitors	Adenocarcinoma	Low
6.1	VEGFR overexpression	VEGFR	VEGFR inhibitors	Small cell carcinoma	Low
6.2	Bcl-2 overexpression	P53/BCL	BCL-2 Inhibitors	Small cell carcinoma	Low
7.1	ROS1 translocation	ROS-1	ROS1 inhibitors	Adenocarcinoma (1.5%)	Medium
8.1	Epigenetic alterations		HDAC inhibitors, epigenetic inhibitors with cytotoxic agents	-	Low
9.1	IGF alterations	IGF	IGF1R monoclonal antibodies, IGF1R TKIs	Adenocarcinoma, Squamous, SCLC	

### Subtype 1

Subtype 1 harbors aberrations in the EGFR gene/pathway – a set of targetable mutations with commercially available inhibitors as well as newer agents on the horizon.

### Subtype 1.1 overview

SUBTYPE 1.1 is characterized by mutations in the EGFR gene that make these tumors responsive to EGFR inhibitors. EGFR encodes a transmembrane receptor that is a member of the ErbB family of tyrosine kinases. EGFR is a cell surface protein that binds to epidermal growth factor and other growth factor ligands to become activated [3]. Upon activation, EGFR tyrosine kinase activity stimulates activation of downstream pathways including Ras/MAPK and Akt/PI3K leading to DNA synthesis and cell proliferation [8], [9], [10], [11].

EGFR has been shown to be dysregulated by various mechanisms in NSCLC, including overexpression, amplification or mutation [12]. NCCN guidelines recommend EGFR testing for the following histologies upon cancer recurrence or metastases: adenocarcinoma (AC) and large cell lung cancers (LC) [13]. EGFR alterations occur in ∼10% of western and ∼50% of Asian patients with lung adenocarcinoma, a histology which comprises 44% of NSCLC cases [14]. Higher EGFR mutation frequency occurs in non-smokers, women, and non-mucinous tumors. Data from several clinical trials has indicated that patients with EGFR mutations have improved progression free survival when treated with EGFR inhibitors compared to patients with the same mutations who received standard-of-care, cytotoxic chemotherapy regimens [5], [15], [16]. The SOE score for determining EGFR mutation status in patients with AC or LC histology is therefore ‘high’.

This subtype includes three classes of mutations: Class I mutations – exon 19 in-frame deletions (44% of all EGFR mutations), Class II - single amino acid changes (L858R 41%, G719 4%, other missense mutations 6%), Class III - exon 20 in-frame duplication/insertions (5%). Eighty-five percent of all EGFR activating mutations are Class I or L858R [17]. EGFR mutations are relatively rare (<3.6%) in squamous cell carcinoma and EGFR testing is not routinely recommended per NCCN guidelines [13], [18].

### Potential therapeutic approach for subtype 1.1

#### EGFR inhibitors

NSCLC large cell and adenocarcinoma patients should be tested at diagnosis for EGFR mutations as those who exhibit such mutations benefit from EGFR inhibitors (e.g. erlotinib or gefitinib) in the first-line setting, as recommended by NCCN guidelines [13]. EGFR inhibitors are playing an increasingly important role in the treatment of NSCLC in select patient groups with aberrantly functioning EGFR and are also used in second- and third-line treatment [4].

#### Combination Therapies

Some additional trials for recurrent or advanced NSCLC are ongoing. These trials will test the efficacy of second-generation EGFR inhibitors or approved EGFR inhibitors (such as erlotinib) in combination with other inhibitor drugs such as MET/VEGFR2 inhibitors.

### Subtype 1.2 – Overview

Subtype 1.2 is defined as NSCLC that harbors a T790M mutation in exon 20 of the EGFR gene. T790M mutations emerge in response to treatment with EGFR TKIs. The T790M mutation accounts for approximately 50% of cases in which acquired resistance to erlotinib or gefitinib occurs [19], [20], [21]. A small percentage of patients may carry the mutation prior to EGFR TKI treatment. Data from clinical trials have indicated the presence of a T790M mutation in EGFR is predictive of resistance to EGFR inhibitors [22]. The SOE score for testing patients with EGFR mutant adenocarcinoma who have developed EGFR TKI resistance is therefore ‘high’.

EGFR TKIs (such as erlotinib or gefitinib) are selective inhibitors of EGFR's kinase domain that work by competing with ATP for binding at the ATP-binding site, thereby preventing autophosphorylation and activation. The T790M mutation affects the gatekeeper residue in the catalytic kinase domain and confers drug resistance by increasing EGFR's affinity for ATP – thus reducing the potency of the ATP-competitive kinase inhibitors [23].

Interestingly, the development of a T790M mutation may actually confer a relatively improved survival, as tumors that acquire it appear to be less aggressive than tumors with EGFR TKI resistance due to other mechanisms [21]. Other aberrations that may give rise to EGFR TKI resistance include PIK3CA mutations, EMT or MET amplification, or conversion to small cell lung cancer histology [3], [24]. Thus, for patients who progress on EGFR TKIs with initial response and known mutation, it would be beneficial to perform a repeat biopsy.

### Potential therapeutic approach for subtype 1.2

Several treatment modalities for addressing drug resistance due to the EGFR T790M mutation are currently being explored.

#### Second Generation EGFR Inhibitors

The first strategy employs second generation TKI's such as afatinib (BIBW2992) that irreversibly inhibits human epidermal growth factor receptor 2 (Her2) and EGFR kinases. Preclinical data have demonstrated that afatinib is a potent irreversible inhibitor of EGFR/HER1/ErbB1 receptors including the T790M variant [25].

#### MET inhibitors

While secondary mutations in EGFR are responsible for the majority of cases of acquired EGFR TKI resistance, activation of other pathways can also lead to resistance. For example, c-MET amplification has been observed in approximately 5%–20% of patients with acquired resistance to EGFR inhibitors. A second therapeutic strategy thus adds various drugs or antibodies capable of inhibiting c-MET (e.g. crizotinib, foretinib, ARQ 197, MetMAb) to first- (erlotinib) or second- (PF-00299804) generation EGFR-TKIs [26], [27], [28]. c-MET is a proto-oncogene that encodes a protein known as hepatocyte growth factor receptor (HGFR) that possesses tyrosine-kinase activity. Recent research has indicated a reciprocal and complementary relationship between T790M and MET amplification. Concurrent inhibition of both may improve patient outcomes [29]. There is also data to support that an antibody against c-MET (MetMAb) is beneficial to patients with high expressing c-MET in combination with erlotinib [30].

#### Hsp90 inhibitors

A third approach involves inhibition of the molecular chaperone Hsp90 with Hsp90 inhibitors such as AUY922, and possibly ganetespib (STA9090). The molecular and cellular consequences of Hsp90 inhibition are not well defined, but some cancers increase levels of active Hsp90, utilizing Hsp90 to process mutant or misexpressed proteins [31], [32]. Hsp90 inhibitors may thus block multiple signaling pathways that are functioning aberrantly in cancer cells [33].

#### Combination Therapies

Some additional trials for recurrent or advanced NSCLC are ongoing. These trials will test the efficacy of various combination therapies including EGFR inhibitors, second- generation tyrosine kinase inhibitors, a dual MET/VEGFR2 inhibitor, a Chk1 inhibitor and more.

### Subtype 1.3 Overview

SUBTYPE 1.3 tumors are defined based on a proteomic signature called VeriStrat, which provides likely responsiveness to EGFR inhibitory therapies such as erlotinib in the absence of EGFR mutations.

VeriStrat utilizes mass spectrometry to evaluate tumor EGFR ligand levels and predict patient response and survival outcome to erlotinib and other EGFR inhibitors from serum samples [34], [35], [36].

VeriStrat profiling is an approved serum analysis diagnostic tool for NSCLC patients who have tested negative for EGFR mutation since some patients with wildtype EGFR status may still benefit from erlotinib treatment regimens. Several recent studies have indicated that VeriStrat classification has significant power to predict response and survival to EGFR inhibitors for several cancer types [34], [35], [36]. The SOE score for VeriStrat classification for patients with wild type EGFR status is ‘high’. One study found that 73% of EGFR wt NSCLC patients previously administered erlotinib had a “VeriStrat good” status. The “VeriStrat good” patients had increased survival after erlotinib treatment compared to the “VeriStrat poor” group [34]. This is thought to be based upon tumor dependence on the EGFR pathway [36].

### Potential therapeutic approach for SUBTYPE 1.3

#### EGFR inhibitors

Patients with a “VeriStrat good” status are predicted to respond well to EGFR inhibitors and should pursue second- and third-line therapy for EGFR positive NSCLC as outlined by several reputable organizations including ASCO and the NCCN [13], [37]. EGFR inhibitors are playing an increasingly important role in the treatment of NSCLC in select patient groups with aberrantly functioning EGFR [3].

#### Second Generation EGFR inhibitors

As noted earlier, the dual EGFR and Her2 inhibitor BIBW 2992 is currently being tested in a phase 2 and 3 clinical trial to explore efficacy in patients with lung adenocarcinoma harboring wildtype EGFR [38].

### Subtype 2

SUBTYPE 2.1 is characterized by mutations in the K-ras gene. K-ras belongs to a family of small GTPases that regulate cellular behavior in response to extracellular stimuli. Ras-regulated signal pathways control processes such as actin cytoskeletal integrity, proliferation, differentiation, cell adhesion, apoptosis, and cell migration via the MAPK and AKT/PI3K pathways [8], [39].

Ras has many isoforms of which K-ras and N-ras and the most relevant to human cancer and are estimated to be mutated in 20–30% of all cancers [40]. While these isoforms are functionally similar, they appear to play unique roles in particular cancers [41]. For example, H-ras aberrations are frequently observed in bladder cancer [42], and N-ras is mutated in approximately 20% of melanomas [43], [44]. K-ras mutations are observed primarily in adenocarcinomas of the lung, colon or pancreas [45].

### Subtype 2.1 overview

Subtype 2.1 is the only subtype in this category and is characterized by mutations in K-ras. K-ras proteins possess intrinsic GTPase activity. Point mutations at codons 12, 13, or 60 in the K-ras oncogene lead to constitutive activation of K-ras protein via changes at the GTP binding domain which prevents the conversion of GTP to GDP [3], [46]. Overall, K-ras mutations have been reported in 15% to 20% of all patients with NSCLC. Approximately 30%–50% of adenocarcinomas are reported to have K-ras mutations, with mutations at codon 12 most commonly detected [3], [14]. K-ras mutations are seen almost exclusively in smokers with one study reporting that 43% of smokers who developed NSCLC had a K-ras mutation compared to 0% of life-long non-smokers with NSCLC [47]. Squamous cell carcinomas are not reported to exhibit K-ras mutations [48].

### Potential therapeutic approach for subtype 2.1

A meta-analysis that included 28 studies of NSCLC reported worse outcomes for patients with K-ras mutations, particularly those with adenocarcinoma histology [46]. K-ras mutations in NSCLC are associated with decreased response to EGFR TKIs. However, K-ras and EGFR mutations are almost always mutually exclusive and the lack of response to EGFR TKIs is most likely due to the absence of an EGFR mutation as opposed to the existence of a K-ras mutation [29]. The SOE score for testing adenocarcinoma patients for K-ras mutations is ‘high’.

#### MEK and mTOR inhibitors

Despite the widespread impact of Ras mutations on cancer, Ras has not been successfully targeted therapeutically in NSCLC. However, several approaches are currently being tested in clinical trials. The first involves concurrently targeting the downstream MAPK and AKT/PI3K pathways [8], [10]. Kinase inhibitors (such as MEK or MTOR inhibitors) that target kinases in pathways downstream of Ras may thus be effective against K-ras mutant cells [49].

#### HSP90 inhibitors

A second approach involves inhibition of the molecular chaperone Hsp90. As noted earlier, HSP90 inhibitors may block multiple signaling pathways that are functioning aberrantly in cancer cells [33].

#### REOLYSIN therapy

REOLYSIN is a proprietary formulation of human reovirus in development by Oncolytics Biotech Inc. for various cancers. REOLYSIN (Reovirus Serotype 3 - Dearing Strain) is a naturally occurring oncolytic virus that preferentially lyses cancer cells, specifically those that have upregulated RAS signaling. The preferential lysis of Ras activated cells and the non-pathogenic nature of the reovirus [50] make REOLYSIN an attractive therapeutic option for Subtype 2.1 K-ras mutant patients. REOLYSIN is in phase 2 clinical trials for NSCLC and phase 1 and 2 clinical trials for multiple other cancers including melanoma, colorectal cancer, osteosarcoma, and more.

### Subtype 3

The EML4-ALK oncogene is a relatively newly-discovered aberration in NSCLC with several targeted agents in active development, including crizotinib which was recently FDA-approved for this subtype [6], [51].

### Subtype 3.1 Overview

Subtype 3.1 is the only subtype in this category and it harbors the EML4-ALK fusion oncogene, a fusion between echinoderm microtubule-associated protein-like 4 (EML4) and anaplastic lymphoma kinase (ALK) [6]. The fusion generates a transforming tyrosine kinase, with as many as nine different variants identified [52]. Several isoforms play a role in lung cancer [53], [54]. EML4-ALK represents a novel molecular target in a small subset of NSCLCs. Patients with EML4-ALK mutant tumors are characteristically younger, female, and never to light smokers [6], [55], [56]. The fusion gene has been observed predominantly in adenocarcinomas (4–7%) and is mutually exclusive with mutations in the EGFR, K-ras, and ERBB2 genes [6], [56]. Interestingly, ALK fusions have also been described in anaplastic lymphomas and in about 50% of inflammatory myofibroblastic tumors (IMTs) with EML4 [3], [57]. The SOE score for testing adenocarcinoma patients for the EML4-ALK translocation is ‘high’.

### Potential therapeutic approach for SUBTYPE 3.1

#### ALK inhibitors

EML4-ALK NSCLC represents a unique subset of NSCLC patients for whom ALK inhibitors have high potential as a very effective therapeutic strategy [6]. Patients who harbor the EML4-ALK fusion do not benefit from EGFR TKIs and should be directed to trials of ALK-targeted therapies [58]. Crizotinib has been approved by the FDA [51], and LDK378 is in development for EML4-ALK NSCLC. Of note, mutations within ALK that lead to crizotinib resistance have already been described, analogous to the T790M mutation leading to erlotinib and gefitinib resistance in EGFR [59].

#### Hsp90 inhibitors

The Hsp90 inhibitor IPI-504 is currently in clinical trials to test its efficacy in lung cancer patients with ALK mutations. As noted earlier, HSP90 inhibitors may block multiple aberrantly functioning signaling pathways in cancer cells [33]. Ganetespib (STA-9090) is also being utilized in clinical trials against ALK translocated lung cancer patients.

### Subtype 4

Subtype 4 harbors aberrations in c-MET. c-MET is a proto-oncogene with important implications in NSCLC [3], [60]. Multiple agents are in active development to target this pathway.

### Subtype 4.1 overview

SUBTYPE 4.1 is characterized by dysregulation of mesenchymal-epithelial transition factor receptor tyrosine kinase (noted as c-MET). c-MET is a proto-oncogene that encodes a tyrosine kinase membrane receptor known as hepatocyte growth factor receptor (HGFR) [60]. Much of c-MET's oncogenic activity stems from its normal physiologic role. In healthy cells c-MET promotes cell growth and cell migration. However, dysregulation of the c-MET pathway leads to cell proliferation, cell survival, angiogenesis, invasion and metastasis [61]. c-MET dysregulation can occur through a variety of mechanisms including c-MET overexpression, activation, overexpression of the c-MET ligand hepatocyte growth factor (HGF), gene amplification, and c-CBL loss of heterozygosity [60], [62]. These alterations, discussed below, are currently grouped together in subtype 4.1. Tests for the detection of all of these abnormalities are not yet considered FDA approved biomarkers, nor is c-MET yet considered a predictive marker that informs clinical decision making for lung cancer patients. Data from clinical trials testing c-MET targeted therapies in genetically selected patient subsets will hopefully increase the SOE score from medium to high in the near future.

c-MET overexpression has been observed in 67% of adenocarcinomas, 57% of large cell carcinomas, 57% of squamous cell carcinomas, and 25% of small cell lung cancers [63]. A recent study demonstrated that adenocarcinoma tumors had a significant increase in the number of MET gene copies indicating that gene amplification is one possible mechanism underlying c-MET overexpression [64]. c-MET activation occurs concomitantly with overexpression and can be detected using immunohistochemical staining for c-MET (called p-MET for detection of phosphorylation typically at sites Y1003 or Y123/1234/1235) [63], [65].

The c-MET ligand HGF can also be overexpressed by tumor cells with moderate expression observed in 45% of lung cancer tumors [66]. When HGF binds to the c-MET receptor it generates MET autophosphorylation leading to activation of the MET pathway enabling cell mobilization and vascularization – two processes that contribute to tumor malignancy [3], [60].

Finally, c-MET dysregulation may also occur through gene mutation. This aberration is clearly distinguishable from the others by gene sequencing [60] and has been assigned to Subtype 4.2.

### Subtype 4.2 overview

Subtype 4.2 is characterized by mutations of c-MET.

c-MET mutations have been observed in both NSCLC and small cell lung cancer. The prevalence of c-MET mutations is relatively low compared to the frequency of c-MET overexpression in lung cancer, but their potential for causing disease progression has been shown to be significant [60]. Mutations are found in two different domains of the c-MET receptor protein. One study of four NSCLC lines and 127 adenocarinomas identified four missense mutations (E168D, L229F, S323G, N375S) in the semaphorin domain, a structural domain of semaphorins, which are a large family of secreted and transmembrane proteins. Another group of mutations were found in the juxtamembrane domain including: R988C and T1010I, S1058P, and an alternative splice variant (lacking the 47-amino acid exon 14) which affects the juxtamembrane domain [63]. Juxtamembrane mutations are significant because this domain is an important regulatory site for the catalytic functions of tyrosine kinases. Mutations in this region resulted in constitutive protein tyrosine phosphorylation and enhanced tumorgenicity in vitro. E168D (sema domain), and R988C and T1010I (juxtamembrane mutations) were also observed in SCLC [66].

Another aberration recently observed to positively correlate with c-MET mutation in NSCLC cell lines is mutation of the Casitas B-lineage lymphoma (c-CBL) gene [62]. c-CBL is a ubiquitin ligase and adaptor ligase that plays a role in normal homeostasis. The high rate of mutation of c-CBL observed in lung cancer is thought to be a combination of somatic missense mutation and loss of heterozygosity (LOH) at the c-CBL locus [3], [60], [62]. c-CBL LOH is associated with mutations in either the MET or EGFR genes as these events have been observed to co-occur in the same samples. It is thus hypothesized that c-CBL mutations may combine with either a MET alteration or EGFR mutation to contribute to lung tumorigenesis and metastasis [62]. Due to a low SOE for c-CBL as a predictive biomarker in the clinical setting, it has not yet been assigned its own subtype.

It should also be noted that MET mutations vary along ethnic and racial lines. One recent study found the highest frequency of c-MET mutations in East Asians. The N375S sema domain mutation was found to be the most frequent mutation overall, occurring more frequently in East Asians than Caucasians, and never in African-American samples [67].

### Potential therapeutic approach for SUBTYPE 4

#### MET inhibitors

Several targeted therapies for the treatment of aberrant MET activity are currently in clinical testing. The first strategy employs direct MET inhibitors capable of interfering with c-MET signaling. Several - including ARQ 197, cabozantinib, and foretinib – are currently in phase 2 and 3 clinical trials.

#### MET antibodies

A single-arm antibody directed against the extracellular domain of MET, called MetMAb, has been tested in a randomized phase II study in patients with previously treated NSCLC with erlotinib. In the intent-to-treat analysis, no benefit was seen with the addition of MetMAb to erlotinib compared to those treated with erlotinib alone. However, in those patients with “MET diagnostic-high” criteria on immunohistochemistry (a composite of intensity and extent of staining that was seen in approximately 50% of patients), there was improvement in progression-free and overall survival, leading to the initiation of a phase III study in this subset of patients [30].

#### HGF antibodies

Two HGF antibodies that target the aberrant action of HGF are currently being tested in lung cancer patients. AMG-102 is a human monoclonal antibody that is in phase I and II clinical trials for NSCLC and several other cancers. Data from one trial in patients with advanced solid tumors suggests that AMG-102 is well tolerated and may inhibit tumor progression in some patients [68]. A second HGF antibody AV-299 is in development by AVEO Pharmaceuticals. Data from phase 1 clinical trials in several cancer types have indicated that AV-299 is tolerable and can be combined favorably with EGFR inhibitors such as erlotinib and gefitinib. Data on the efficacy of AV-299 in treatment of NSCLC should be available toward the end of 2011.

#### Hsp90 Inhibitors

Another strategy involves inhibition of the chaperone protein, Hsp90, which is required for c-MET protein (and other kinase) folding and stability [3]. AUY922 and IPI-504 are currently in phase 1 and 2 clinical trials for lung cancer.

### Subtype 5

Subtype 5 harbors aberrations in the AKT/PI3K pathway. PI3K acts antagonistically with the lipid phosphatase, PTEN, to tip the balance between two signaling molecules, PIP2 and PIP3 [69], [70]. Upon growth factor stimulation, PI3KCA is activated increasing PIP3 levels which drives phosphorylation of AKT and downstream processes such as higher protein translation, cell division and reduced apoptosis [10], [69].

### Subtype 5.1 overview

Subtype 5.1 is characterized by aberrations in PI3K, a lipid kinase that regulates growth in the AKT/PI3K pathway. The PI3K protein family is divided into three classes and several subclasses based on primary structure, regulation, and in vitro lipid substrate specificity. Of these the Class Ia is the most studied, partly because of its role in cancer. These proteins are composed of a catalytic subunit known as p110 and a regulatory subunit known as p85. To date the PI3K is the only family member found to have somatic mutations in human cancer. These mutations occur predominantly in the helical or kinase domains of the alpha p110 catalytic subunit which is encoded by the PI3KCA gene [71].

The PI3K/AKT pathway plays a key role in regulating mammalian cell proliferation and survival. Activating mutations in PI3KCA have been implicated in various cancers including melanoma, breast cancer, colorectal cancer and NSCLC. In lung cancer, PI3KCA gene amplification occurs at a much higher frequency than do activating mutations [69], [71]. According to one study, PI3KCA copy number gain was found in ∼33% of squamous cell tumors, ∼6.2% of adenocarcinomas, and ∼2% of SCLC tumors. Mutations were found in 3.6% of SCC, 2.6% of AC, but not in SCLC [71]. Exon 9 was most frequently mutated site in AC and SCC. Exon 20 was also affected, though more frequently in AC than in SCC [18], [71]. The SOE score for genetic testing for PI3K aberrations in lung cancers is currently ‘low’ since only data from pre-clinical models are available.

### Potential therapeutic approach for SUBTYPE 5.1

There are three potential targets for therapeutic intervention in this pathway: AKT, PI3K, and mTOR. There are several drugs in clinical development against all three targets and a few drugs against mTOR that are currently approved for other cancer types.

#### PI3K inhibitors

Several phase I and II trials are currently ongoing targeting PI3K in NSCLC either alone or in combination with standard, cytotoxic chemotherapy. These agents include XL765 – which also inhibits mTOR – BKM120, and GDC-0941.

#### AKT inhibitors

Phase I and II trials are currently recruiting that offer MK-2206 – an oral, potent, allosteric inhibitor of AKT – in combination with either erlotinib or gefitinib in advanced NSCLC.

#### mTOR inhibitors

Multiple phase I and II trials are recruiting patients for treatment of advanced NSCLC with mTOR inhibition either alone or in combination with chemotherapy or radiation therapy. These include agents such as everolimus, sirolimus, and temsirolimus as well as agents that have dual mTOR and PI3K activity such as XL-765.

### Subtype 5.2 Overview

Subtype 5.2 harbors inactivating mutations or deletions in PTEN, a lipid phosphatase that negatively regulates growth through the AKT/PI3K pathway, as noted above [69], [70].

Inactivation of PTEN is associated with a variety of cancers including glioblastoma, melanoma, prostate, breast, endometrial cancers, and NSCLC. Loss of the PTEN tumor suppressor results in tumorigenesis. PTEN mutations (in exons 5–8) have been observed in 8% of SCC and 1% of adenocarcinomas [72]. The SOE score for genetic testing for PTEN aberrations is currently ‘low’ since only data from pre-clinical models are available.

### Potential therapeutic approach for Subtype 5.2

Same as seen for Subtype 5.1.

### Subtype 6

This subtype is characterized by aberrations in the vascular endothelial growth factor (VEGF) pathway. The VEGF pathway regulates vascular angiogenesis. Tumors usurp this pathway to promote self survival and proliferation. Downstream of VEGF, B-cell lymphoma 2 (Bcl-2) is an anti-apoptotic regulator protein that has been implicated in a number of cancers including SCLC. VEGF can promote the survival of tumor cells through the induction of Bcl-2 expression [73] which in turn can induce VEGF expression in certain tumor types [74], [75], [76].

### Subtype 6.1 overview

SUBTYPE 6.1 is characterized by overexpression of vascular endothelial growth factor receptor (VEGFR). VEGFR is the receptor for vascular endothelial growth factor (VEGF), an important signaling protein involved in vasculogenesis and angiogenesis. Aberrations in the VEGFR pathway contribute to the rapid growth and high metabolic activity characteristic of SCLC and are associated with poor outcome for this cancer type [77]. The SOE score for genetic testing for VEGFR aberrations in SCLC is currently ‘low’ since only data from pre-clinical models are available.

### Potential therapeutic approach for Subtype 6.1

#### VEGFR inhibitors

Current trials evaluating VEGFR inhibition in SCLC include the agents vandetanib and sunitinib, either in combination with cytotoxic chemotherapy or as maintenance therapy. To date, trials done in unselected patients have been disappointing. Future biomarker directed trials based upon serious biologic rational are needed to determine the true efficacy of these inhibitors in select patient groups.

### Subtype 6.2 overview

Subtype 6.2 is characterized by aberrations in Bcl-2, a key inhibitor of cell apoptosis. The Bcl-2 family of proteins contains both pro- and anti-apoptotic members which regulate apoptosis via a delicate balance. The Bcl-2 pathway is activated in response to cellular stress such as growth factor deprivation, hypoxia, cell detachment or DNA damage via p53.

Several lines of evidence point to a key role of Bcl-2 in SCLC pathogenesis:

1. Bcl-2 has been reported to be up-regulated in 73%–90% of human SCLC tumors [78], [79], [80].
2. Anti-sense suppression of Bcl-2 leads to decreased survival of SCLC cell lines and increased sensitivity to chemotherapy [81].
3. Targeted inhibition of Bcl-2 using small molecule inhibitors killed SCLC cell lines treated in vitro and caused regression of established tumors in xenograft models (mice) [82], [83], [84].

The SOE score for genetic testing for Bcl-2 aberrations in SCLC is currently ‘low’ since only data from pre-clinical models are available.

### Potential therapeutic approach for Subtype 6.2

Several drugs that could provide therapeutic relief to this subtype of patients, including YM155, ABT-737, AT-101 and TW37, are in the early stages of development. The drug classes discussed here are also good candidates for targeted therapies for SCLC patients.

#### Bcl-2 inhibitors

One anti-apoptotic agent, oblimersen, an anti-sense agent targeted at nuclear Bcl-2 has exhibited mixed results for SCLC. A small phase I study reported a promising response rate of 83% (10 of 12 evaluable patients) for oblimersen with paclitaxel in patients with chemorefractory relapsed SCLC, based on observations that Bcl2 family members contribute to paclitaxel resistance. A follow-up phase II study tested carboplatin and etoposide with or without oblimersen and the addition of oblimersen neither improved patient response rate nor survival [84], [85].

#### Hedgehog inhibitors

Phase I and II trials treating SCLC with Hedgehog inhibition alone or in combination with standard cytotoxic chemotherapy are currently ongoing. These agents include GDC-0449 and XL-139.

#### Picornavirus

Picornaviruses are non-enveloped, positive-stranded RNA viruses with an icosahedral capsid. One particular picornavirus, Seneca Valley Virus-001, is a biologic agent which may have anti-neoplastic activity on its own. A phase II trial is currently ongoing in which patients with extensive-stage SCLC are randomized to Seneca Valley Virus-001 versus placebo after induction cytotoxic chemotherapy.

### Subtype 7

The recent discovery of ROS-1 mutations has shown that drugs such as crizotinib may have more activity than the already known inhibition of the EML4-ALK translocation and c-MET.

### Subtype 7.1 overview

SUBTYPE 7.1 harbors the ROS-1 translocation. Patients with ROS-1 mutant tumors are characteristically young, non-smokers. The fusion gene has been observed predominantly in adenocarcinomas (∼1.5%) and is mutually exclusive with mutations in the EGFR, K-ras, and EML4-ALK genes [56]. The SOE score for testing adenocarcinoma patients for the EML4-ALK translocation is ‘medium’.

### Potential therapeutic approach for SUBTYPE 7.1

#### Crizotinib

ROS1 NSCLC represents a unique subset of NSCLC patients for whom the c-MET/ALK inhibitor crizotinib has shown potential as a very effective therapeutic strategy. As noted earlier, the ALK inhibitors crizotinib and LDK378 are currently in development for EML4-ALK NSCLC though at present, only crizotinib has been shown to be effective in treating this small subset of patients with a ROS1 translocation [54]. A phase I trial evaluating crizotinib in advanced solid tumors which possess the ROS1 translocation is currently ongoing.

### Subtype 8.1 overview

Subtype 8.1 involves epigenetic alterations. Epigenetics is the study of reversible changes to DNA and the nucleosome that affect expression of genes and can be pharmacologically manipulated. The earliest recognized of these changes was DNA methylation. In tumors, it was found that methylation at the 5 position of cytosines in the context of CpG islands (areas in the promoter of genes with a relative over-abundance of cytosine-guanine dinucleotides) led to decreased expression of affected genes, often tumor suppressor genes or other growth regulatory molecules. This process is complex and is controlled by DNA methyltransferase inhibitors, DNMT1, DNMT3a and DNMT3b. Hypermethylation of promoter regions in cancer, specifically lung cancer is an almost universal finding, with differences in different types of cancer [86]. Methylation inhibitors, typically nucleotide analogues were found to be capable of inhibiting DNA methyltransferases, demethylating DNA and leading to gene re-expression. Several nucleoside analogues, 5-azacytidine and 5-aza, 2′-deoxycytidine have been found to be effective in the treatment of mylodysplastic syndromes and leukemias and are FDA approved for these indications [87].

Control of gene expression is more complex such that in addition to DNA methylation, changes in the nucleosomal structure via post-translational modifications of histones, specifically methylation, acetylation, phosphorylation and sumoylation at the amino terminus of histone H3 and histone H4 can also lead to alterations of gene expression. The interplay between DNA methylation and histone post-translational modifications is complex, and includes histone methyltransferases and acetyltransferases as well as deacetylases. Typically, more heavily acetylated histone tails leads to a more “open” chromatin configuration and is more accessible to transcription factors and the basal transcriptional machinery. In vitro experiments have shown that histone deacetylase inhibitors (therefore leading to more heavily acetylated histones) leads to greater expression of target genes, and several HDAC inhibitors have been evaluated in clinical trials showing activity particularly in cutaneous T cell lymphoma [88]. Combination therapy with methylation inhibitors and HDAC inhibitors has attraction given that a number of preclinical in vitro experiments have demonstrated dramatic synergistic effects in terms of gene expression as well as other cellular effects when these epigenetic modifying agents are combined [87], [89]. Recent experiments have demonstrated that HDACs affect the acetylation status of more than histones, and that a number of other important cellular proteins are also subject to these post-translational modifications. These proteins include tubulin, p53, E2F and others. The relevance of these proteins in the effect of HDAC inhibitors on tumor cells has not been fully evaluated.

### Potential Therapeutic Approach to SUBTYPE 8.1

A number of trials have attempted to utilize epigenetic therapy including DNA methylation inhibitors, HDAC inhibitors, the combination of methylation inhibitors and HDAC inhibitors as well as epigenetic agents combined with standard cytotoxic agents. One of the great questions in epigenetic therapy is whether there are specific patients who are more likely to benefit from these therapeutic agents.

### Subtype 9

This subtype is characterized by aberrations in the insulin-like growth factor (IGF) axis. This axis involves two receptors (IGF1R and IGF2R), their ligands (IGF-1 and IGF-2), and multiple binding proteins. It is theorized that defects in the IGF axis are involved in the development of multiple malignancies, including NSCLC and SCLC [90].

### Subtype 9.1 overview

The IGF axis is important in normal cells for differentiation, metabolism, and growth with features of endocrine, paracrine, and autocrine control. Alterations in this pathway can lead to uninhibited proliferation, playing an important role in the development of neoplasia [90]. Expression of IGF1R in particular appears to be a negative prognostic indicator – at least on univariate analysis – and is seen in up to 31% of patients with NSCLC [91], [92]. The SOE score for testing for IGF aberrations or IGF levels in lung cancer, however, is currently ‘low’ as much of the data regarding this pathway is pre-clinical while several clinical trials evaluating inhibition of this pathway have been negative or are still ongoing [93], [94].

### Potential therapeutic approach for SUBTYPE 9.1

There are several therapeutic approaches to inhibiting the IGF axis using monoclonal antibodies against the extracellular domain of IGF1R as well as small-molecule inhibitors of the intracellular tyrosine kinase domain of the same receptor [93], [94], [95].

#### Monoclonal antibodies

Multiple monoclonal antibodies have been investigated in the treatment of lung cancer. One such antibody, figitumumab, was shown to improve response rates in both adenocarcinoma and squamous cell histologies when combined with carboplatin and paclitaxel in a phase II trial [95]. However, the phase III trial was suspended due to increased deaths and toxicity from heart failure and myocardial events in those who received figitumumab [93]. The combination of erlotinib with the IGF1R monoclonal antibody, R1507, did not reveal a statistically significant improvement in PFS or OS in NSCLC [94] though investigations with another IGFR1 monoclonal antibody, cixutumumab, in the treatment of SCLC and NSCLC are currently ongoing [96], [97].

#### IGF1R tyrosine kinase inhibitors

OSI-906 is a selective small molecule, dual kinase inhibitor of both IGF1R and the insulin receptor. A phase II clinical trial combining OSI-906 with erlotinib in NSCLC is currently recruiting patients [98], [99].

## Discussion

Lung cancer treatment has traditionally been viewed within the bounds of a ‘one-size-fits-all’ approach with the use of cytotoxic chemotherapy. With such poor outcomes, it is no wonder that a sense of nihilism has pervaded the management of these patients over the last half-century. During the past decade, however, research has shown that lung cancer is a molecularly-complex amalgam of diseases with specific derangements leading to vastly different outcomes and, more importantly, the chance to develop effective, targeted treatment [3]. This paper represents an outline of a ‘molecular disease model’ for lung cancer as was previously performed by our group for melanoma [7]. The purpose of this paper is to highlight the key molecular aberrations observed in lung cancer, including those for which there is still only preclinical data, with the hope of accelerating the process of translational research by providing a dynamic conduit between basic science researchers, clinical trialists, and treating physicians.

Currently, EGFR and K-ras mutational testing are already common practices for most community oncologists [13] while testing for the EML4-ALK translocation has recently become a regular phenomenon outside of only tertiary, academic oncology centers due to the FDA-approval of crizotinib [6], [51]. The development of effective chemotherapeutics which target the EGFR pathway – erlotinib and gefitinib – took place over 15 years while the targeting of the EML4-ALK translocation with crizotinib has progressed steadily in less than a third of that time [4], [5], [6]. These targets that are actionable today are observed primarily in adenocarcinoma. However, as researchers become more adept at identifying key molecular changes and developing targeted therapies for these mutations, the momentum of the development process will only continue. This will allow for the development of therapies that target the other aberrations discussed here, including those pathways relevant to other NSCLC histologies and SCLC. The result will be the effective management of patients based on the molecular profiles of their cancers.

This review has focused on the treatment of lung cancer based on the targeted treatment of single gene or pathway alterations. Some of these targets are actionable today. This is highlighted by the online lung cancer therapy finder application that can be accessed online (www.collabrx.com/lung), and is based upon the lung cancer molecular disease model discussed here. By entering the detailed characteristics of a patient's tumor such as stage, histology, and previously performed molecular testing, detailed information can be generated including what is currently known about this tumor subtype, what further molecular testing is recommended, next steps in the management of said tumor, and if any clinical trials are available for those patients who exhibit these specific mutations. This is a powerful tool for physicians and patients alike allowing for truly personalized care in an easy-to-use and regularly-updated format.

## Methods

The initial subtypes and associated practice guidelines defined here were identified by consensus of a panel of recognized lung cancer experts, and supported by detailed analysis of the peer-reviewed scientific literature. Subtypes are defined based on the status of key lung cancer genes/pathways and their combinations. Each subtype is defined by one key oncogene/tumor suppressor (such as EGFR for subtypes 1.1 to 1.3, and K-ras for subtype 2.1), either by itself or in combination with others that play a supportive role (such as EGFR activating mutations in exons 19 and 21 and the exon 20 resistance mutation T790M in the case of subtypes 1.1, 1.2, and 1.3).
